# Bioluminescent Orthotopic Mouse Models of Human Localized Non-Small Cell Lung Cancer: Feasibility and Identification of Circulating Tumour Cells

**DOI:** 10.1371/journal.pone.0026073

**Published:** 2011-10-11

**Authors:** Pierre Mordant, Yohann Loriot, Benoit Lahon, Yves Castier, Guy Lesèche, Jean-Charles Soria, Marie-Catherine Vozenin, Charles Decraene, Eric Deutsch

**Affiliations:** 1 INSERM U1030 & Université Paris XI, Institut Gustave Roussy, Villejuif, France; 2 Service de Chirurgie Thoracique & Université Paris VII, Hôpital Bichat, AP-HP, Paris, France; 3 Département de Médecine, SITEP& Université Paris XI, Institut Gustave Roussy, Villejuif, France; 4 Département de Recherche translationnelle, Institut Curie, Paris, France; 5 CNRS UMR144, Paris, France; 6 Département de Radiothérapie & Université Paris XI, Institut Gustave Roussy, Villejuif, France; Penn State Hershey Cancer Institute, United States of America

## Abstract

**Background:**

Preclinical models of non-small cell lung cancer (NSCLC) require better clinical relevance to study disease mechanisms and innovative therapeutics. We sought to compare and refine bioluminescent orthotopic mouse models of human localized NSCLC.

**Methods:**

Athymic *nude* mice underwent subcutaneous injection (group 1-SC, n = 15, control), percutaneous orthotopic injection (group 2-POI, n = 30), surgical orthotopic implantation of subcutaneously grown tumours (group 3-SOI, n = 25), or transpleural orthotopic injection (group 4-TOI, n = 30) of A549-luciferase cells. Bioluminescent *in vivo* imaging was then performed weekly. Circulating tumour cells (CTCs) were searched using Cellsearch® system in SC and TOI models.

**Results:**

Group 2-POI was associated with unexpected direct pleural spreading of the cellular solution in 53% of the cases, forbidding further evaluation of any localized lung tumour. Group 3-SOI was characterized by high perioperative mortality, initially localized lung tumours, and local evolution. Group 4-TOI was associated with low perioperative mortality, initially localized lung tumours, loco regional extension, and distant metastasis. CTCs were detected in 83% of *nude* mice bearing subcutaneous or orthotopic NSCLC tumours.

**Conclusions:**

Transpleural orthotopic injection of A549-luc cells in *nude* mouse lung induces localized tumour, followed by lymphatic extension and specific mortality, and allowed the first time identification of CTCs in a NSCLC mice model.

## Introduction

Non-small cell lung cancer (NSCLC) is the leading cause of cancer-related mortality worldwide. Despite numerous clinical trials of preclinically promising drugs, no major outbreak has been made in NSCLC management in the last decades. Although there is strong evidence based medicine supporting the clinical use of cytotoxic, around 3 types of agents have shown consistent but limited efficacy (platinum, taxanes, gemcitabine). More recently, targeted therapies have waived important hopes given their ability to directly hit cancer cell specific survival mechanisms. In sharp contrast with the robustness of the underlying biological rational, clinical translational of this knowledge into clinical benefit still remains challenging since targeted therapies showed only a marginal survival benefit when considering the whole NSCLC patients population [1]–[4]. After these initial trials, retrospective molecular analyses of tumor tissue led to the definition of subgroups of patients that favourably respond to investigational treatments, with management of certain cancers being revolutionized [5]. Among patients with NSCLC who receive erlotinib, the presence of an EGFR mutation increased responsiveness to the agent [6], [7], but concerned only 16% of patients [8]. More recently, the inhibition of anaplasic lymphoma kinase (ALK) has been reported to result in tumour shrinkage or stable disease in most patients with NSCLC harbouring EML4-ALK fusion genes, but concerned only 2 to 7% of NSCLC [9]. However, in the meantime, considerable efforts have been spent in clinical trials testing active treatments on resistant tumors [2], [3], [10]–[12].

The discrepancy between the values of the clinical rationale, the amount of preclinical data on one hand and the low output of their transfer in clinical trials suggest that the relevance of preclinical models may be questioned. Especially, preclinical testing should have a more clearcut positive versus negative predictive value [13]. Current in vivo preclinical models are not reflecting the steps of malignant progression from normal tissue to precancerous lesion and then to cancer (localized and then metastatic). Ideal model should mimic the natural history of human cancer, in order to improve our understanding of cancer pathogenesis, predict the efficacy of the investigated treatment and identify which treatment will fit to which patient before the design of clinical trials. In this context, xenografts models remain the cornerstones of preclinical experiments and will gain from which technical improvements, along with the recent development of computer-assisted drug design, computer modeling, and genetically engineered animals [14]. Human NSCLC xenografts may be implanted into immunodeficient animals either subcutaneously (SC) or orthotopically. In one hand, SC xenografts of lung cancer are easy to perform and to follow, but lack relevance regarding (i) the natural history of cancer due to absence of lymphatic or metastatic extension, and (ii) the prediction of drug efficacy in clinical trials as witnessed by the high proportion of negative clinical trials [13]. In the other hand, orthotopic models of lung cancer are technically more challenging, do not always mimic the natural history of lung cancer, but raise considerable hope regarding drug screening [15]. The recent increase in our understanding in the cross talk of the tumor stroma [16]–[18] and its involvement in drug resistance, metastasis, immune escape, angiogenesis strongly suggests that orthotopic models should provide valuable information that one could not obtain from sub-cutaneous models.

Considering thoracic tumors, the technical challenge is to grow “lung tumor cells within the chest”. Several techniques may be used to this aim, including intra tracheal administration [19] or percutaneous orthotopic injection [20] of NSCLC cells in solution, and surgical orthotopic implantation of subcutaneously grown tumour originating from NSCLC cell line [21]. Intratracheal administration is associated with diffuse initial spreading of the cells to both lungs, interfering with progression and lung metastasis process, therefore favouring percutaneous and surgical models. However, technical feasibility and natural history of these two models have never been compared. This study sought to compare and refine these two orthotopic xenografts models of lung cancer, with the ultimate goal to reproduce human NSCLC progression, from initially localized intraparenchymatous tumor, to metastasis development and general worsening. For this purpose, we compared four xenografts models, including SC injection used as control, percutaneous orthotopic injection and surgical orthotopic implantation as previously described, and transpleural orthotopic injection as a new model. To allow longitudinal follow up of tumor progression and further evaluation of treatment efficacy, we used a cell line transfected with luciferase and performed *in vivo* bioluminescent imaging. Feasibility was assessed by perioperative mortality and engraftment rate. Clinical relevance was assessed by initial intra parenchymatous location, loco-regional extension, metastatic extension, median survival and histological aspect. In an exploratory analysis, identification of Circulating tumor cells (CTCs) was performed using the Cellsearch® assay (Veridex LLC, USA).

## Materials and Methods

### Cell line

**Figure 1:**
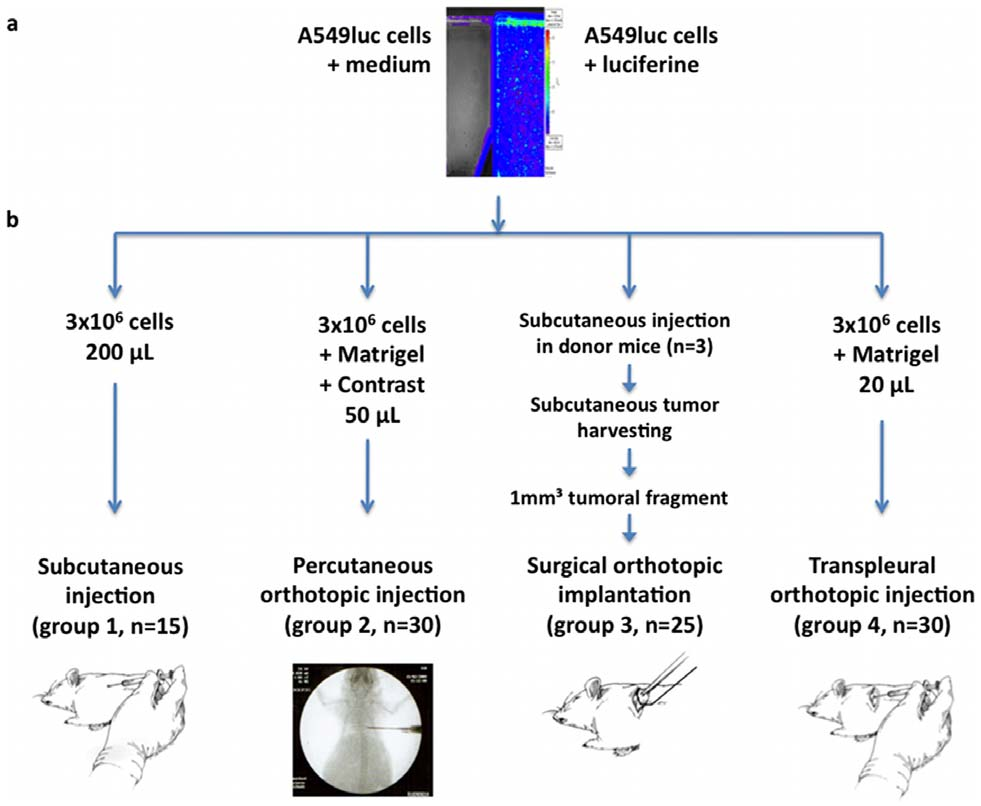
Study flowchart. a. A549 cells stably transfected with luciferase were tested by direct application of serum saline (left side, control) or luciferine (right side), and analysed after optical imaging (Time 1 min after addition of luciferine, exposure 1 min, bin medium). b. Animals were divided in 4 groups, subcutaneous injection (group 1-SC), percutaneous orthotopic injection under radiographic control (group 2-POI), surgical orthotopic implantation after orotracheal intubation and thoracotomy approach (group 3-SOI), and transpleural orthotopic injection after costal layer surgical exposure (group 4-TOI).

Human A549 lung adenocarcinoma cell line stably transfected with luciferase (A549luc) was purchased from Caliper Lifesciences Corp. Cells were cultured in complete medium, consisting of 10% (v/v) fetal bovine serum, 2 mmol L-glutamine, and 50 units/mL penicillin-streptomycin in RPMI (all from Invitrogen). Cells were grown at 37°C and 5% CO2 in an incubator, tested as mycoplasm free using a PCR mycoplasma detection kit (MycoProbe, R&D Systems, MN, USA), and tested as luciferase positive using direct application of 2 mL of a 150 µg/ml solution of luciferine (Firefly Luciferin, Caliper Lifescience Corp, USA), followed by immediate bioluminescent imaging (IVIS system, Caliper Lifescience Corp, Figure 1a).

### Animals

Six-week-old female athymic mice were purchased from Janvier (Elevage Janvier, Mayenne, France), and kept in appropriate conditions. Once arrived in our animal facility, mice were divided in 4 groups, underwent xenografts implantation following the protocols described below (Figure 1b), and subsequent bioluminescent imaging. Per procedure mortality rate, engraftment rate, locoregional and metastatic progression were determined. The end of the experiment was defined as cachexia, dyspnea, or clinical worsening. Animals were then humanely killed. Primary tumour, lungs, and mediastinal lymph nodes were surgically harvested, fixed using a formalin-free, water-based concentrate (Finefix, Milestone S.r.l., Sorisole, Italy), and embedded in paraffin. Histological examination was then performed. Cancer-related mortality was assessed. All animal experiments were approved by the local Ethics Committee (CEEA IRCIV/IGR n°26, registered with the French Ministry of Research), and were in compliance with the European Directive 86/609/CEE and French laws and regulation.

### Subcutaneous (SC) injection (group 1 - SC)

A solution of 3×10^6^ A549luc cells in 200 µL of culture medium was injected subcutaneously into the left flank region of the animals (n = 15), using 1-mL tuberculin syringes with 30 G hypodermic needles (Becton Dickinson, NJ, USA).

### Percutaneous orthotopic injection (group 2 - POI)

Animals (n = 30) were anesthetized using isoflurane and putted in a position of dorsal decubitus. Under radiographic control, 3×10^6^ A549luc cells in a solution containing 30 µL of culture medium, 10 µL of contrast medium (Omnipaque 300, GE Healthcare SA, France), and 10 µL of mouse sarcoma extracellular matrix (Matrigel, BD Biosciences, NJ, USA) were injected percutaneously into the left lung of the animals using 1-mL tuberculin syringes with 30 G hypodermic needles (Becton Dickinson, NJ, USA). Mice were then allow to rest on a heating carpet until fully recover.

### Surgical orthotopic implantation (group 3 - SOI)

As a first step, 3×10^6^ A549luc cells were injected subcutaneously into the flank region of 3 donor animals. Once SC tumours reached 50 mm3, tumours were harvested and subdivided into 1 mm^3^ pieces constituting tumour grafts. Recipient animals (n = 25) were anesthetized using intraperitoneal injection of xylazine (20 mg/kg) and ketamine (100 mg/kg), intubated using a 22 G i.v catheter, mechanically ventilated (stroke volume 200 µL, respiration rate = 120 strokes/min, Ventilator Minivent Type 845, Harvard Apparatus, MA, USA) and putted in a position of right lateral decubitus. A 2-cm skin incision was made below the left scapula and a sharp dissection of the chest muscles was performed, in order to expose the costal layer. A 0.5 cm intercostals incision between the third and fourth costa on the chest wall was made, and the chest wall was opened. The left lung was taken up by a forceps, clamp with a carotid clamp (Scanlan International, MN, USA), incised with surgical knife, and the tumour was introduced promptly into the lung parenchyma. The incision of the lung parenchyma was closed with surgical glue (Bioglue, Gamida, France). The incision of the chest wall was closed by a 4-0 polypropylene suture (Prolene, Ethicon Inc, USA). Mice were then allow to rest on a heating carpet until fully recover.

### Transpleural orthotopic injection (group 4 - TOI)

Animals (n = 30) were anesthetized using intraperitoneal injection of xylazine (20 mg/kg) and ketamine (100 mg/kg), and putted in a position of right lateral decubitus. A 2-cm skin incision was performed below the left scapula, and a sharp dissection of the chest muscles was performed, in order to expose the costal layer. On observing the left lung motion through the pleura, 3×10^6^ A549luc cells in a solution containing 10 µL of culture medium, and 10 µL of mouse sarcoma extracellular matrix (Matrigel, BD Biosciences, NJ, USA) was directly injected through the intercostal space into the lung to a depth of 3 mm using a 29 G needle permanently attached to a 0,5 mL insulin syringe (Becton Dickinson, NJ, USA). The skin incision was closed by a 4-0 polypropylene suture (Prolene, Ethicon Inc, USA). Mice were then allow to rest on a heating carpet until fully recover.

### Bioluminescent imaging

Bioluminescent imaging was detected from luciferase expressing A549 cells (A549luc) after implantation of the xenografts into mice. Luciferin (Firefly Luciferin, Caliper Lifescience Corp, USA) was used as the substrate for the luciferase expressing tumour cells and injected intra peritoneally at a concentration of 150 mg/kg in PBS, 15 minutes before imaging. Mice were then anesthetized using 2% isofluorane and imaged using a cooled CCD camera (IVIS system, Caliper Lifesciences Corp, USA). Exposure times ranged from 1 minute to 1 second. Images were quantified as photons/s using the Living Image software (Caliper Lifesciences Corp, USA). Bioluminescent imaging was performed at day one, then weekly, and then immediately before sacrifice. Xenograft implantation rate was defined as the number of primary tumour on imaging 2 weeks after grafting divided by the number of animal alive after the procedure. Subsequent locoregional extension, lymphatic and haematogenous metastasis rates were determined during a 2-month follow up, and confirmed by pathologic examination at the end of the experiments.

### Identification of CTCs

To further characterize SC and transpleural injection models, we sought to identify CTCs in these models, using 2 additional groups. In the CTC group 1, athymic *nude* mice underwent SC injection of A549luc cells in both flanks (n = 6). In the CTC group 2, animals underwent general anesthesia, chest wall incision, and transpleural injection of cells in the parenchyma of the left lung (n = 15). After 2 weeks, bioluminescent imaging was performed, and tumour-bearing animals were identified. During the third week, 6 tumour-bearing animals from each group were randomly chosen and anesthetized. A venous blood puncture of 600 µL was performed in the cavernous sinus and was tested for CTC using the CellSearch® system and a modified protocol based on the CellSearch® Epithelial Cell kit (Veridex LLC, USA). Briefly, to identify human CTCs in mouse blood, each 600 µL mice blood samples was mixed with 7 mL of healthy human blood. Then, each sample was automated enriched for cells expressing the epithelial-cell adhesion molecule (EPCAM) with antibody-coated magnetic beads, and cells were labeled with the fluorescent nucleic acid dye 4′,6-diamidino-2-phenylindole dihydrochloride (DAPI). Fluorescently labeled monoclonal antibodies specific for leukocytes (CD45–allophycocyan) and epithelial cells (cytokeratin 8,18,19-phycoerythrin) were used to distinguish epithelial cells from leukocytes. Cell Enrichment and labeling were performed using the CellSearch® Autoprep. The identification and enumeration of CTCs was performed using the CellSearch® Analyzer II. CTCs were defined as nucleated cells lacking CD45 and expressing cytokeratin 8, 18, 19. As a negative control, a solution containing 600 µL of non-tumor bearing mouse blood and healthy human blood was analysed. As a positive control, 50 and 500 A549luc cells were analysed in a solution containing 600 µL of medium and 7 mL of healthy human blood.

### Statistical analysis

Normally distributed continuous variables were expressed as means ± standard deviation, and compared with unpaired t-tests. Categorical data were expressed as counts and proportions, and compared with Fisher exact tests. All tests were 2-sided and a p-value<.05 was considered significant. All data analyses were performed with the free R software (http://www.r-project.org, R Foundation for Statistical Computing, Vienna, Austria).

## Results

### Feasibility

Feasibility was assessed by determination of perioperative mortality and engraftment rate. The perioperative mortality was null in group 1-SC used as a control, and significantly higher in group 3-SOI (60%, p<0.001) but not in groups 2-POI (6%, p = .79) or 4-TOI (3%, p = .72). Assessing the effect of a learning curve on the perioperative mortality of group 3-SOI, we observed a non significant decrease in the mortality between the first 10 animals and the former 15 animals (mortality 80% vs 47%, respectively, p = .21). No significant difference was observed between groups 2-POI and 4-TOI regarding the perioperative mortality rate (p = .99, **Table 1**). The engraftment rate was 100% in group 1-SC used as a control, and significantly lower in group 2-POI (68%, p = .037), group 3-SOI (60%, p = .034) and group 4-TOI (65%, p = .027). No significant difference was observed between the 3 experimental groups regarding the engraftment rate (p = .90, **Table 1**).

**Table 1:** Per-procedure mortality, tumor engraftment, and tumor extension rates.

	Number of animals(n)	Per-procedure mortality rate(n)	Implantation rate(n)	Loco-régional invasion(n)	Metastasis(n)
**Subcutaneous injection (group 1)**	15	0	100% (15)	-	-
**Percutaneous orthotopic injection (group 2)**	30	6% (2)	68% (19)	53% (15)	3% (1)
**Surgical orthotopic implantation (group 3)**	25	60% (15)	60% (6)**	-	-
**Transpleural orthotopic injection (group 4)**	30	3% (1)	65% (19)	7% (2)	3% (1)*

* Bone metastasis.

** n = 1 false positive (infection); n = 2 false negatives.

### Clinical relevance

**Figure 2:**
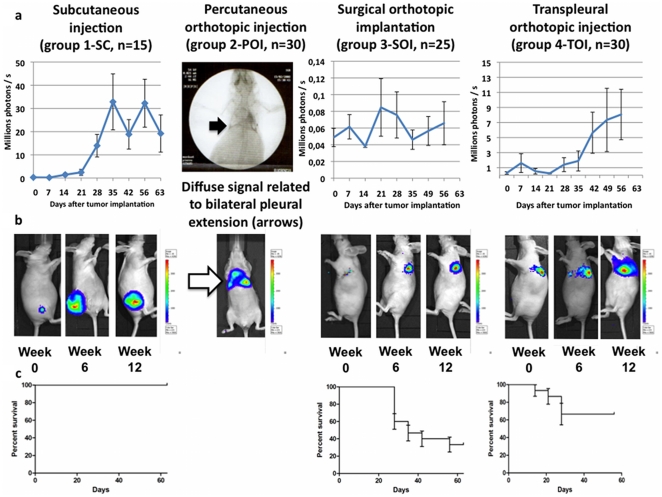
Follow-up using bioluminescent imaging and survival curves. a. Evolution of the photons count (expressed as 10^6^ photons/s) over time, from day 0 (implantation) to day 63 (end of the bioluminescent follow up). For each time, the mean and standard deviation of the animals alive are reported. In group 2-POI, percutaneous injection led to pleural seeding in 53% of the cases, as postoperative X-ray showed contrast agent in either left pleural space, right pleural space (anatomical continuum), or both. b. Representative evolution of the bioluminescent signal, from day 14 to day 42. In group 2-POI, pleural seeding created a signal located at the right basis of the thorax, contradicting with the primary goal of this study. No subsequent follow-up as been performed for this group. c. Survival curve from the time of tumor implantation to 2 months using the Kaplan-Meier method and 95% confidence interval bars. The 2-month survival is 100% in group 1-SC, 40% in group 3-SOI, and 65% in group 4-TOI.

**Figure 3:**
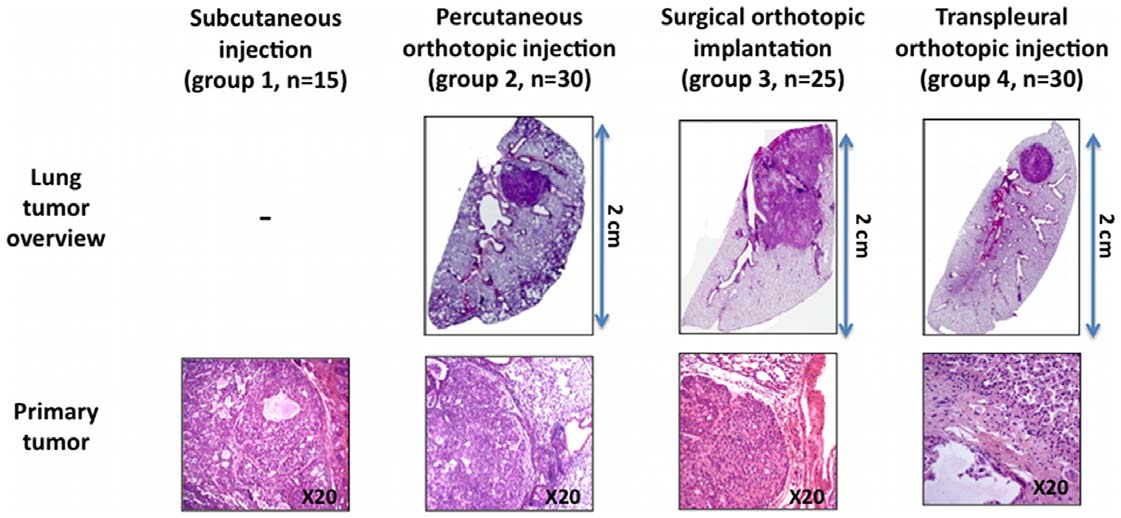
Pathological examination. Surgical orthotopic implantation (Group 3) resulted in localized infiltrating tumors, whereas percutaneous orthotopic injection (Group 2) and transpleural orthotopic injection (group 4) resulted in localized rounded nodules. Histological examination revealed a high proportion of undifferentiated carcinoma in group 1-SC tumor, but a low proportion in the 3 experimental groups. Furthermore, the 3 experimental groups were associated with more capsule rupture and vascular emboli than group 1-SC.

Clinical relevance was assessed by location of the initial tumor, imaging of tumor extension, survival analysis and histological examination. The initial tumor was confined to the SC tissue in group 1-SC and to the lung parenchyma in group 3-SOI. In group 2-POI, 53% of the animals underwent pleural seeding during percutaneous orthotopic injection and developed initial loco regional extension to both pleurae. As the goal of this study was to obtain an intrapulmonary localized NSCLC model, this immediate pleural seeding disqualified the technique. In group 4-TOI, the tumour was initially confined to the lung parenchyma (**Table 1** **,** **Figure 2a and 2b**). Bioluminescent imaging found irregular curves in group 1-SC and 3-SOI, with high inter individual and chronological variations. No animal developed locoregional or metastatic extensions during follow up. In group 2-POI, no bioluminescence curve was plotted because of perioperative pleural seeding. One animal developed bone metastasis. In group 4-TOI, bioluminescent imaging found a regular, exponential curve after day 21. Seven percents of the animals developed locoregional extension to homolateral pleura as an evolution of the primary tumour. One animal developed bone metastasis (**Table 1** **,** **Figure 2a**). Survival analysis revealed that median survival was not achieved after a follow up of 2 months in group 1-SC and 3-SOI. In group 2-POI, no survival curve was plotted because of perioperative pleural seeding. In group 4-TOI, median survival was 40 days (**Table 1** **,** **Figure 2c**). Histological examination revealed SC round tumours with a regular shape, high proportion of undifferentiated carcinoma, few capsular ruptures, and few vascular or lymphatic emboli in group 1-SC. In group 2-POI, regular intraparenchymatous tumors were obtained, with a low proportion of undifferentiated carcinoma, a high number of capsular ruptures, and vascular or lymphatic emboli. In group 3-SOI, irregular intraparenchymatous tumors were obtained, with a low proportion of undifferentiated carcinoma (+), capsular ruptures (++) and a high number of vascular or lymphatic emboli. The findings were similar in group 4-TOI and group 2-POI (**Figure 3** **,** **Table 2**).

**Table 2:** Pathological examination and CTC levels.

	Primary tumor	Undifferenciated population	Capsular rupture	Emboli	CTC
**Subcutaneous injection (group 1)**	SC	+++	+	+	
**Percutaneous orthotopic injection (group 2)**	Lung+pleura	+	+++	+++	**2.5±1.76/animal**
**Surgical orthotopic implantation (group 3)**	Lung	+	++	+	
**Transpleural orthotopic injection (group 4)**	Lung	+	+++	+++	**9.5±9.35/animal**

### Identification of CTCs

**Figure 4:**
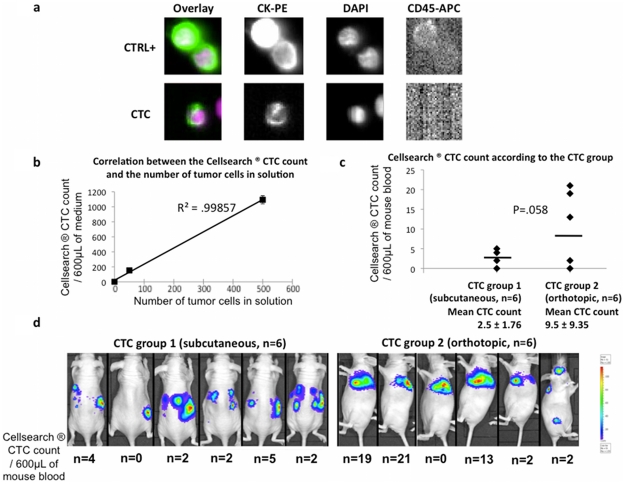
Identification of circulating tumor cells. a. Representation of the staining of circulating cells using the Cellsearch® assay. CTC are defined as CD45−, DAPI+, and CKPE+ cells. - Upper line: as a positive control, 50 and 500 A549luc cells were analysed in a solution containing 600 µL of medium and 7 mL of healthy human blood. Here, as an example, both CD45−, DAPI+, CKPE+ cells are tumor cells. - Bottom line: representation of the results of the experimental groups. Here, as an example, this CD45−, DAPI+, CKPE+ cell is a CTC. b. Correlation between the Cellsearch ® CTC count and the number of tumor cells in solution. As controls, 0 (negative control), 50 and 500 (positive controls) A549luc cells were analysed in a solution containing 600 µL of medium and 7 mL of healthy human blood. This experiment demonstrated a correlation between the Cellsearch® CTC count and the number of tumor cells in solution (R^2^ = .99857). c. Number of CTC according to the experimental group, CTC group 1 (SC) or CTC group 2 (orthotopic). The overall positivity of the Cellsearch ® assay is comparable in both groups (83%), with a trend toward more CTC in the orthotopic rather than in the subcutaneous model. d. Representation of the bioluminescent signal and number of CTC detected in each of the 12 animals.

Xenograft implantation rates were 100% in CTC group 1 (SC, n = 6) and 60% in CTC group 2 (TOI, n = 9). Three Cellsearch® assays were performed in each control group, and 6 Cellsearch® assays were performed in each experimental group. Negative controls demonstrated zero or one CD45−, DAPI+, CKPE+ cell per assay (data not shown). Positive controls demonstrated a correlation between the number of tumor cells in solution and the Cellsearch® CTC count (**Figure 4a and 4b**). In CTC group 1-SC, 5 assays (83%) were positive for CTCs detection (CTCs level range 2–5, mean 2.5±1.76). In CTC group 2-TOI, 5 assays were positive for CTCs detection (CTCs level range 2–21, mean 9.5±9.35). All together, 3 weeks after tumour implantation, CTCs were detected in 83% of *nude* mice bearing either SC or orthotopic NSCLC tumours (**Figure 4c and 4d**). When comparing groups, the difference in CTC levels showed a trend toward statistical significance (p = .058, **Figure 4c** **,** **Table 2**).

## Discussion

### Tumor implantation site and method impacts tumor development and kinetics

Comparing SC, percutaneous orthotopic injection and surgical orthotopic implantation models with intend to refine the follow up of tumour progression using bioluminescent imaging, we faced the limits of these models, including organ discrepancy (SC), frequent pleural seeding (percutaneous orthotopic injection) and high perioperative mortality (surgical orthotopic implantation). Furthermore, none of these models allow a reliable follow up of tumour progression using bioluminescent imaging. Therefore, we developed a transpleural orthotopic injection model, combining low perioperative mortality, reasonable engraftment rate, scarcity of pleural seeding, and tumour progression with metastasis for which tumor progression may be monitored by bioluminescent imaging. Furthermore, this study allows identification of CTCs in murine models of NSCLC for the first time to date. We believe that besides functional imaging, the application at the preclinical in vivo stage of CTCs will contribute to define biomarkers of tumour burden and treatment efficacy in subsequent clinical trials.

### SC xenografts models

SC xenografts from human-derived cell lines to immunodeficient animals have been the gold standard of preclinical cancer experiments for decades [14]. This model presented several advantages, including technical feasibility (absence of anesthesia, easy-to-perform injection) and tumor accessibility (direct measurement of the SC tumor allowing longitudinal follow up). However, SC models are limited by discrepancy between xenograft origin and host microenvironnement, including extra cellular matrix, paracrine signals, and therefore do not model Paget's “seed and soil” theory [22]. This discrepancy has been found to impact tumour response to cytotoxic agents and radiation therapy [23], [24] and certainly impact the response to novel anticancer agents. This discrepancy might be of critical importance in NSCLC, were hypoxia and neoangiogenesis play a major role in tumor progression [25], and occur differently in SC and intrathoracic xenografts [26]. Last, only a minority of SC models disseminate and progress to the metastatic stage, the absence of progression from localized to metastatic stage through loco-regional lymph node extension is therefore a major limitation of SC models while the major cause (2/3) of cancer death is metastatic disease.

These differences have major implications regarding tumor progression, with few circulating tumor cells and no metastasis in our experiments, pointing a major limit to drug evaluation, with two risks. The first risk is to push into the clinic a molecule or a drug combination with little or no efficacy. As an example, the combination of chemotherapy and EGFR inhibitors has proved efficacy in SC models of NSCLC [27], [28], but has never reached significant efficacy in clinical trials including unselected NSCLC patients upon EGFR status [3], [10], [29]. The second risk is to stop the development of promising drugs. As an example, HIF-1alpha antagonist PX-478 showed no measurable activity against NSCLC SC xenografts, but inhibited progression and spread of orthotopic human small cell lung cancer and lung adenocarcinoma in mice, and is finally tested in phase 1 trial [30].

Altogether, these data suggest that SC xenografts are not fully adequate for the preclinical study of NSCLC, and should only be used and analyzed carefully. Similar conclusions have been drawn from SCLC preclinical drug testing [31].

### Previous orthotopic models

To take the influence of organ specificity and tumor micro environnement into account, orthotopic models have been progressively developed over the last 20 years, with the ultimate goal to obtain a single intraparenchymatous lung tumor that mimic the clinical situation and allow longitudinal follow up. Intravenous [32], intrabronchial [33]–[35] and intrapleural administration [36], [37] of lung cancer cell lines resulted in pleural, locally advanced, or multiple synchronous tumors. Therefore, these models may be interesting to study these particular situations, but may not be extended to localized intrapulmonary NSCLC, constituting the majority of NSCLC patients and the basis of further tumor progression.

Genetically engineered mouse models (GEMMs) have also been developed to study NSCLC in mice. These animals are usually p53 mutated, favoring genetic instability and tumor formation, and develop lung tumors driven by the EGFR [38], KRAS [39], PIK3CA [40], BRAF [41] oncogenic activation or the presence of EML4-ALK fusion oncogene [42]. KRAS mutated lung cancer may be observed in mouse strains carrying oncogenic alleles of KRAS that can be activated only on a spontaneous recombination event in the whole animal [43], or after KRAS sporadic activation through Cre-lox mediated somatic recombination after adenoviral mediated delivery of Cre recombinase in lung epithelia [39]. Both models allow the development of KRAS-mutated lung cancer in a short period of time. However, these models are limited by their distance to clinical situation, as mutations of one or two oncogenes or tumor suppressor genes are far from the multi step progression of lung cancer occurring on underlying chronic inflammation. Furthermore, GEMM models result in hundreds of primary NSCLC in the same period of time those are difficult to follow and to quantify during preclinical testing and non-invasive imaging. Despite these drawbacks, GEMM have the advantage to refine the definition of oncogenes and to take immune response into account. Recent studies have reported that GEMM may be of interest when studying the efficacy of common NSCLC treatments [44].

Surgical implantation of NSCLC fragment into the lung of immunodeficient mice via thoracotomy was first reported in 1992 [21], [45], [46]. Tumor fragment came from primary fresh tumors or subcutaneously-grown tumors developed after NSCLC cells injection. Tumor fragment were inserted into the lung parenchyma, on the visceral pleura, or on the parietal pleura, according to the disease extension aimed for. Per procedure mortality was below 10%, engraftment rates ranged between 60 and 100%, but not systemic metastasis was observed. The same model was then developed in rat [47] and confirmed using primary cultured cells from surgical-resected NSCLC in SCID mice [48]. Addition of GFP to implanted tumor allow in vivo imaging of orthotopic xenografts development, and metastatic extension to mediastinal lymph nodes, controlateral lung, and bone [49]–[52]. However, these experiments are technically difficult, require specialized laboratory and dedicated manpower, and despite encouraging preliminary results [53], have not been generalized in preclinical NSCLC research to date.

### Orthotopic transpleural injection combined to bioluminescent imaging

Orthotopic injection of NSCLC cells in nude mice has been developed to overcome the technical difficulties of thoracotomy [20], [54], [55]. This technique is technically feasible with a low mortality rate and a high engraftment rate, with 2 limits. The first limit is the impossibility to predict perioperatively if the tumor will be localized to the lung parenchyma or if there is a pleural seeding leading to a locally advanced tumor. Therefore, to obtain a single tumor limited to the lung parenchyma, we used a high number of cells in a small volume of medium and add some matrix of mouse sarcoma, improving the solution anchorage to the lung parenchyma. We injected this solution trans pleurally, under direct vision of the lung motion through the intercostal space, combining technical feasibility (absence of orotracheal intubation and mechanical ventilation) and high precision.

The second limit is the requirement of a longitudinal follow up using micro CT in the absence of fluorescent or bioluminescent imaging. MicroCT has limited spatial resolution and is available only in a limited number of facilities. Therefore, we used luciferase-transfected NSCLC cell line to allow in vivo bioluminescent imaging. This technique has already been reported in a percutaneous orthotopic model with pleural seeding [56], and has now been applied successfully to transpleural model with localized intra parenchymatous NSCLC model.

This xenogenic, orthotopic and bioluminescent model allowed the first time identification of CTCs in murine models of NSCLC. CTCs are released into the bloodstream from tumors of epithelial origin [57].

As haematogenous dissemination of tumor cells is the main mechanism for distant metastasis, the assessment of cancer patients' blood is a highly desirable approach for detecting systemic tumor cell spreading [58] and residual disease. CTCs may be identified directly after automatic enrichment and immunocytochemical detection (CellSearch® System) [59], magnetic bead enrichment and laser scanning cytometry (Maintrac System) [60], microfluidic harvesting and molecular characterization (CTC-Chip) [61], or membrane filter device (Isolation by Size of Epithelial Tumor cells – ISET) [62]. The CellSearch® system is the most available, and is now FDA-approved in breast, colorectal, and prostate cancers. This new application to NSCLC preclinical model could be of significant interest to study the role of CTCs in NSCLC progression and response to new therapeutic strategies.

### Study limits and future developments

Our study has important limitations regarding the use of immunodeficient animals and the heterogeneous genetic background of the xenografts, as the validity of xenograft models remains disputed by the compromised of anti tumour immunity and the absence of clearly characterized molecular abnormalities in comparison with GEMM. Therefore, we believe that the next step refinement in preclinical models in vivo could take advantage of the *in situ* implantation of tumour fragments or cells into the lung as well as the advantage of murine tumour models (immune response and GEMM derived tumours).

The application of current NSCLC treatments to both models would ultimately determine the most relevant model for preclinical studies. The value and the additional benefit of these novel approaches will have to be compared not only to other techniques available but also in terms of go/no go decision making value toward the clinical stage. Our vision is that functional imaging and biological pre and per treatment parameters such as CTCs may also contribute to demonstrate the improvement in clinical value of these approaches. In particular, we propose that orthotopic implantation of GEMM derived lung tumours into the lung could be an optimal strategy to evaluate tumour immunity and to monitor the efficacy of therapies at the non metastatic, locally advanced tumour stage (i.e. surgery and/or radiotherapy combined to novel agents).

### Conclusion

The tumour microenvironment plays a major role in promoting tumour growth [63]. In xenografts models, tumour localization is important regarding sensitivity to chemotherapy [23], and will be critical regarding sensitivity to targeted therapies, as suggested by the involvement of integrin in the interactions between the tumour and its environment, and subsequent cross talks with targeted pathways. For these reasons, we have developed a murine intrapulmonary model of human non-small cell lung cancer, associated with low peri operative mortality, high engraftment rate, locoregional grow, and development of metastasis. This model allows the first time identification of a high number of circulating lung tumour cells in mice bearing human xenografts of NSCLC.
